# Rapid detection of fentanyl, fentanyl analogues, and opioids for on-site or laboratory based drug seizure screening using thermal desorption DART-MS and ion mobility spectrometry

**DOI:** 10.1016/j.forc.2017.04.001

**Published:** 2017-04-27

**Authors:** Edward Sisco, Jennifer Verkouteren, Jessica Staymates, Jeffrey Lawrence

**Affiliations:** National Institute of Standards and Technology, Materials Measurement Science Division, Gaithersburg, MD, USA

**Keywords:** Fentanyl, Trace detection, IMS, DART-MS, Opioids, Narcotics

## Abstract

Fentanyl and fentanyl analogues represent a current and emerging threat in the United States as pure illicit narcotics and in mixtures with heroin. Because of their extreme potency, methods to safely and rapidly detect these compounds are of high interest. This work investigates the use of thermal desorption direct analysis in real time mass spectrometry (TD-DART-MS) and ion mobility spectrometry (IMS) as tools for the rapid and sensitive (nanogram to picograms) detection of fentanyl, 16 fentanyl analogues, and five additional opioids. Competitive ionization studies highlight that detection of these compounds in the presence of heroin is readily achievable, down to 0.1% fentanyl by mass with TD-DART-MS. With IMS, detection of nanogram levels of fentanyl in a binary fentanyl and heroin mixture is possible but can be complicated by decreased resolution in certain commercial instrument models. Modifications to the alarm windows can be used to ensure detection of fentanyl in binary mixtures. Additionally, three complex background matrices (fingerprint residue, dirt, and plasticizers) are shown to have a minimal effect of the detection of these compounds. Wipe sampling of the exterior of bags of questioned powders is shown to be a safe alternative method for field screening and identification, removing the need to handle potentially lethal amounts of material.

## 1. Introduction

Opioid abuse is a growing epidemic that creates unique challenges for law enforcement, first responders, and medical personnel. Fentanyl and fentanyl analogues are many times more potent than morphine, making accidental exposure through the seizure of powders and pills life-threatening. The Drug Enforcement Administration (DEA) issued a warning to law enforcement in June of 2016 to exercise extreme caution when handling possible fentanyl-containing materials [1]. The lethal dose of fentanyl can be as low as 2 mg [1], with ingestion, inhalation, and absorption through the skin as possible exposure routes. Carfentanil, commonly used as a large animal tranquilizer, is present in illicit U.S. drug markets, is approximately 100 times more potent than fentanyl, and has been used as a chemical warfare agent in anti-terrorist activities [2]. The Centers for Disease Control (CDC) has issued a health alert on the rise of unintentional overdoses as a result of fentanyl and fentanyl analogues in the U.S. from clandestinely produced and trafficked fentanyl (commonly referred to as non-pharmaceutical fentanyl (NPF)) in the form of counterfeit pills and heroin adulterants [3].

Field testing that requires direct handling of suspected drug powders is discouraged due to safety concerns, as materials must be treated as potential NPF, both in the field and in the lab, until a qualitative analysis can be performed. This extended period of uncertainty about the contents of the suspicious material is undesirable, as it requires extreme vigilance during handling. A rapid, safe screening test for fentanyl and fentanyl analogues that is successful in realistic sample scenarios (i.e. mixtures with heroin) would protect emergency and laboratory personnel and could provide valuable medical treatment information. Current colorimetric techniques require macroscopic amounts of material, making them unsuitable for safe field testing of NPF. Ion mobility spectrometry (IMS) is a field-deployable technology useful for qualitative screening of street drugs and is sensitive to nanogram levels of fentanyl, even in the presence of heroin [4]. The contamination on bag exteriors may be sufficient for detection, providing necessary information regarding the bag’s potential contents and what precautions should be taken. What is unknown is the specificity of IMS to detect a wide range of fentanyl analogues, including carfentanil, particularly in the types of mixtures expected in street drugs or when contaminated with dirt and fingerprint residues from the outside of bags.

Another potential field deployable, and laboratory-based, screening technique is thermal desorption direct analysis in real time mass spectrometry (TD-DART-MS), which has been demonstrated for direct analysis of drugs collected onto wipes without the traditional solution-based sample preparation [5]. TD-DART-MS is sensitive to picogram levels of a wide range of illicit drugs [5], and the ability of this technique to detect fentanyl and fentanyl analogues has not been reported to our knowledge. The use of traditional DART-MS has also been extensively reported in literature [6–10]. Additionally, the configuration of the TD-DART-MS system is arranged to minimize potential exposure to the analyst because of aersolization and/or vaporization. TD-DART-MS instruments have potential applications in mobile laboratories, emergency vehicles, and hospitals. The CDC advises two-tiered testing to identify the specific fentanyl compound on which individuals are suspected to have overdosed; the identity of the compound is essential information for understanding and controlling outbreaks [3]. Current guidelines recommend an enzyme-linked immunosorbent assay (ELISA) screen for fentanyl followed by gas chromatography/mass spectrometry (GC/MS) analysis. As NPF concentrations in blood can be quite low, a wipe-based technique such as TD-DART-MS targeting solid trace contamination on the individual or their belongings may be a more effective approach. TD-DART-MS may also be useful in emergency medicine, providing a rapid identification of the specific NPF to make informed choices about treatment. The common antidote for opioid-related overdoses, naloxone, may not be effective in single doses against the more potent NPFs such as carfentanil and other synthetic opioids such as U-47700 [11].

This study provides some of the necessary data needed to support the use of IMS and TD-DART-MS for applications in the law enforcement, emergency, and medical communities. Fentanyl, sixteen different fentanyl analogues, and five opioids (heroin, U-47700, buprenorphine, methadone, and naloxone, which is found in combination with buprenorphine) were evaluated for detection by IMS and TD-DART-MS, to determine spectral characteristics and limits of detection. Selected compounds were evaluated for changes in sensitivity and selectivity of detection when combined with heroin and/or excipients, and against background collected from sampling the outside of bags, fingerprints, and dirt. The potential for false negatives or false positives for NPF using IMS was evaluated.

## 2. Materials & methods

### 2.1. Materials

A total of 22 compounds were analyzed, as listed in Table 1. These compounds included fentanyl, fentanyl analogues, and five additional commonly abused opioids. All fentanyl analogues were purchased as hydrochloride salts from Cayman Chemical (Ann Arbor, MI, USA)^*^ in powdered form or, when possible, as 1 mg mL^−1^ methanolic solutions. Fentanyl was purchased in powder form as fentanyl citrate (US Pharmacopeia, Rockville, MD, USA) and the remaining opioids were purchased as 1 mg mL^−1^ methanolic solutions from Cerilliant (Round Rock, TX, USA). Powdered samples were dissolved gravimetrically in methanol (Chromasolv^®^ grade, Sigma-Aldrich, St. Louis, MO, USA), and all samples were further diluted in methanol. Solutions were then pipetted or inkjet printed [12,13] (additional parameters in the Supplemental information) onto *meta*-aramid fiber wipes (Nomex^®^, Smiths Detection, Hemp-stead, UK) for IMS analysis, or polytetrafluoroethylene (PTFE)-coated fiberglass wipes (DSA Detection, North Andover, MA, USA) for TD-DART-MS analysis. The five excipients examined (acetaminophen, caffeine, mannitol, quinine, and procaine) were purchased in powder form (Sigma-Aldrich) and dissolved in methanol. Materials used as background simulants include an artificial fingerprint material [14] and a dirt simulant (NIST SRM 1944). Polyethylene glycol – 600 (PEG-600) diluted in methanol was used as the mass tuning compound for the mass spectrometer of the TD-DART-MS system.

### 2.2. TD-DART-MS

A thermal desorption direct analysis in real time mass spectrometer (TD-DART-MS) configuration was used in this work and included a thermal desorption unit independent of the DART ionization source. The details of the configuration are discussed elsewhere [5], but briefly, the system consists of a traditional DART source and Vapur^®^ interface (IonSense, Saugus, MA, USA) with a glass T-junction installed in between the source and the interface. A thermal desorption unit (Morpho Detection, Newark, CA, USA) was mounted to the T-junction, perpendicular to the source and interface. Parameters for this portion of the system included 400 °C DART gas stream temperature, 255 °C thermal desorption unit temperature, and Vapur^®^ flow rate of 3.8 L min^−1^ (±0.1 L min^−1^). After evaporation of the solvent, prepared PTFE wipes were inserted into the thermal desorption unit. The TD-DART system was interfaced to a JEOL JMS-T100LP time-of-flight mass spectrometer (JEOL USA, Peabody, MA, USA). Relevant mass spectrometer parameters included: positive ion detection, +400 V peaks voltage, 100 °C orifice temperature,+5 V orifice 2 and ring lens voltage, 2300 V detector voltage, and a scan range of 60–700 *m*/*z* at 1 scan s^−1^. The orifice 1 voltage was switched between +20 V, +30 V, +60 V, and +90 V to evaluate fragmentation spectra. Unless otherwise stated, the data presented was collected using a +30 V orifice 1 voltage. The instrument was tuned using PEG-600 in the range of 70–700 *m*/*z*.

### 2.3. IMS

A dual tube IMS (Ionscan 500 DT, Smiths Detection) was used for this work. Prepared meta-aramid wipes containing dried deposits were inserted directly into the thermal desorber unit of the instrument. Tube 1 was configured to detect positive ions (narcotics), and was used exclusively for the data presented herein. The data from tube 2, which was set to detect negative ions (explosives), was not used. The instrument configuration included a desorber temperature of 245 °C, a drift tube temperature of 244 °C, and an inlet temperature of 265 °C. Data was collected for a total of 8 s by sampling full spectra at 30 discrete intervals over that time. A detection algorithm was used to analyze each plasmagram in the group of 30 for peaks within defined detection windows, as the shape and position of peaks can change during the collection time. Most of the compounds evaluated in this study have not been studied previously by IMS, therefore reduced mobility (K_0_) values were measured for the most prominent peaks from replicate samples using instrument firmware for peak fitting that references an internal calibrant of nicotinamide (K_0_ = 1.8600). Nicotinamide also serves as the reactant ion peak (RIP), which is a constant background spectral component. For selected compounds, measured K_0_ values were used to create detection windows in the instrument library, using a minimum peak intensity detection threshold of 50 counts to trigger an alarm. In addition to the newly added NPF alarms, a large suite of drug alarms was left active, which included: heroin, methamphetamine, amphetamine, cocaine, 3,4-methylene dioxymethamphetamine (MDMA), hydrocodone, oxycodone, and morphine.

## 3. Results & discussion

### 3.1. Representative response and sensitivity

**Fig. 1:**
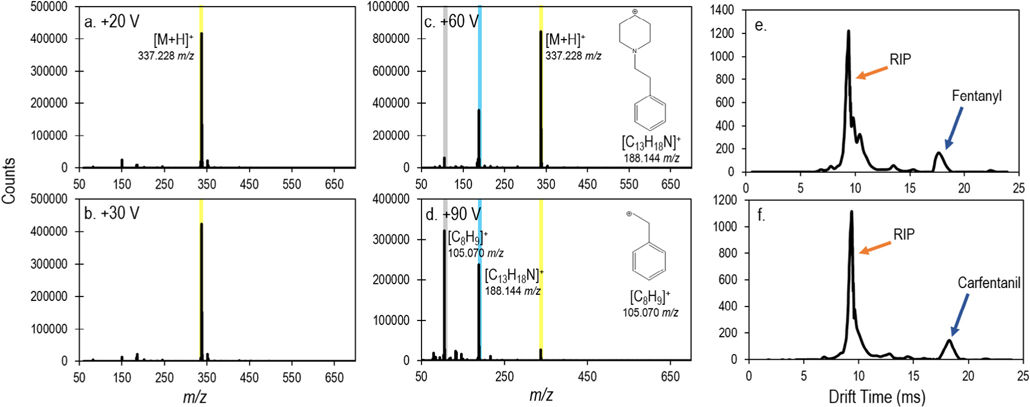
(a–d) Representative TD-DART-MS mass spectra of a 100 ng deposit of fentanyl at the four orifice 1 voltages studied. Representative IMS plasmagrams of 10 ng fentanyl (e) and carfentanil (f) deposits. The reactant ion peak (RIP) is also identified.

To minimize the risk of exposure for first responders and forensic scientists, it is crucial that detection of fentanyl and fentanyl analogues be completed with a high degree of specificity and sensitivity, while facilitating sampling from the residue on the outside of a container, not the bulk powder. To evaluate the abilities of the TD-DART-MS and IMS instruments to meet these requirements, representative spectra and sensitivities of the 22 compounds were obtained. Representative spectra using TD-DART-MS were obtained by pipetting 100 ng deposits onto the PTFE wipes and directly inserting them into the thermal desorption unit. All compounds were analyzed at four orifice 1 voltages (+20 V, +30 V, +60 V, and +90 V) to establish fragmentation pathways and be consistent with data compiled in the NIST DART Forensics Library [15]. All compounds readily produced signals differentiable from the background, an example of which is shown in Fig. 1 (additional spectra Figs. S1–S17). As has been exhibited with other narcotics [5], responses of the opioids and fentanyl analogues were dominated by protonated molecular ions at low orifice 1 voltages of +20 V and +30 V (Fig. 1 and Table 1), with increasing fragmentation observed at the higher (+60 V and +90 V) orifice 1 voltages. Fentanyl and fentanyl analogues followed similar patterns of fragmentation, with the nitrogen-carbon bond being the predominant fragmentation point. At the low fragmentation voltage (+30V) there were three sets of fentanyl analogues that produced identical protonated molecular ion peaks: 1) acetyl and benzyl, 2) o-fluorobutyryl and p-fluoroisobutyryl, and 3) butyryl, isobutyryl, and trans-3-methyl fentanyl. Analysis of these compounds at the higher fragmentation voltages revealed that isomeric compounds (i.e. butyryl and isobutyryl fentanyl) were unable to be differentiated because of identical fragment ions. If isomeric information was required, it may be able to be achieved using an IMS-MS configuration. Non-isomeric compounds, however were differentiated based on their fragmentation spectra (i.e. acetyl fentanyl has a base peak of 105.070 *m*/*z* at 90 V while benzyl fentanyl had a base peak of 91.055 *m*/*z*). Fragmentation patterns of other opioids were more unique: the loss of a hydroxide ion was commonly observed for naloxone and buprenorphine; the loss of the nitrogen containing end group was observed with methadone and heroin; and fragmentation across the carbon-nitrogen bond in U-47700 was observed (Table 1, Fig. S17).

In addition to identifying the representative spectra, the instrument sensitivities for detection of these compounds were also established. Limits of detection (LOD_90_) were determined in accordance with ASTM E2677 (Standard Test Method for Determining Limits of Detection in Explosive Trace Detectors) [16] in which four levels around a presumptive LOD are analyzed (10 replicates/level) in addition to 10 replicates of the blank. From the ASTM Standard a LOD_90_ corresponds to the lowest mass which can be detected off of a wipe with a 90% probability of true detection.

The levels of all compounds except heroin evaluated for TD-DART-MS were (0, 0.5, 1.0, 5.0, and 10.0) ng wipe^−1^, while heroin analyzed at (0, 1.0, 2.0, 10.0, and 20.0) ng wipe^−1^. Table 1 shows the calculated LOD_90_ for the compounds examined. For all compounds, with the exception of heroin and buprenorphine, sub ng wipe^−1^ detection limits were obtained, with most fentanyl analogues detectable at or below a few hundred picograms wipe^−1^.

Determination of representative IMS responses were completed by analyzing 1 ng to 100 ng of the compounds deposited individually on meta-aramid wipes. In most cases, the spectra were simple combinations of the RIP and one distinct peak for the compound, as represented by fentanyl and carfentanil in Fig. 1e and f. Shapes and locations of additional fentanyl analogue peaks are shown in Fig. S18. The primary peak detected for each compound is likely to be a product ion of M^+^ or [M+H]^+^, and the reduced mobilities are linearly correlated with molecular weight (Fig. S18). There are five pairs of fentanyl analogues that cannot be differentiated on this instrument on the basis of K_0_ (differences of less than ±0.002). These pairs are: 1) acetyl and benzyl, 2) p-fluoroisobutyryl and furanyl, 3) butyryl and isobutyryl, 4) isobutyryl and trans-3-methyl, and 5) 4-methoxy and valeryl fentanyl. In addition to the fentanyl analogue overlaps, despropionyl fentanyl and naloxone could not be differentiated on the basis of K_0_. Since IMS is based on cross-sectional area in addition to molecular mass, the technique was able to differentiate one set of isomeric compounds that the TD-DART-MS could not (o-fluorobutyryl and p-fluoroisobutyryl). Of the 22 compounds listed in Table 1, none have overlaps in K_0_ with the illicit drugs already active in the instrument library, and no false positive alarms for these drugs were observed.

LOD_90_ values were determined for a small subset of compounds on IMS (fentanyl, buprenorphine, heroin, methadone, and naloxone), as shown in Table 1, with sensitivities roughly equal to those obtained using TD-DART-MS. For the remaining compounds approximate sensitivities are reported, and were calculated by finding the lowest deposited mass that produced a peak capable of being fit to a Gaussian function, with a height of at least 50 counts, across three replicates. These values are also shown in Table 1. For all compounds analyzed by TD-DART-MS and IMS, sensitivities were at or below 10 ng wipe^−1^, highlighting the possibility of detection by swiping the outside of a bag, or other surface, instead of needing to sample bulk powder.

### 3.2. Competitive ionization in binary mixtures

Though it is useful to demonstrate low level detection of these compounds, it is more important to evaluate whether or not these compounds can be detected in the presence of other drugs or excipients. Since fentanyl is commonly encountered in the United States as a minor component in a mixture with heroin [17], it is necessary to establish whether or not the heroin or any excipients will inhibit detection of fentanyl. In order to evaluate whether detection of fentanyl would be possible in more realistic settings, a series of experiments were carried out to establish whether signal suppression would occur in the presence of heroin or common adulterants (mannitol, acetaminophen, quinine, procaine, and caffeine). The excipients investigated were chosen based on data suggesting they are common in heroin [18].

**Fig. 2:**
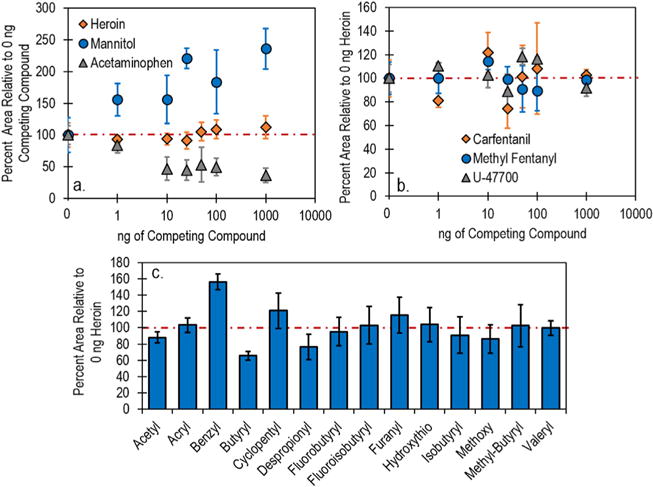
(a) Competitive ionization studies using TD-DART-MS of fentanyl in the presence of increasing amounts of heroin, mannitol, and acetaminophen. (b) Competitive ionization studies of carfentanil, methyl fentanyl, and U-47700 in the presence of increasing amounts of heroin. (c) Competitive ionization study of the remaining fentanyl analogues in the presence of 100 ng heroin. Error bars represent the standard deviation of 5 (a and b) or 3 (c) replicate measurements.

To evaluate the effect of competitive ionization in binary mixtures using TD-DART-MS, increasing amounts of heroin, or an excipient, were added to 1 ng wipe^−1^ deposits of fentanyl. Levels of the “competing compound” (heroin or the excipient) were 0 ng wipe^−1^ (just fentanyl analyzed), 1 ng wipe^−1^, 10 ng wipe^−1^, 25 ng wipe^−1^, 50 ng wipe^−1^, 100 ng wipe^−1^, and 1000 ng wipe^−1^ to obtain competing compound: fentanyl ratios of 0:1, 1:1, 10:1, 25:1, 50:1, 100:1, and 1000:1. Once analyzed, the extracted ion chronograms of the fentanyl molecular ion (337.228 *m*/*z*) were used to calculate peak areas. The response of fentanyl in the binary mixture relative to pure fentanyl was then calculated by plotting the peak area in the mixture relative to peak area of pure fentanyl (Fig. 2).

Fig. 2 highlights the effects of three of the compounds – heroin, mannitol, and acetaminophen – on the fentanyl signal. In these plots, the dotted line represents the response of 1 ng wipe^−1^ deposits of fentanyl. Points above the dotted line indicate the competing compound enhanced the response of fentanyl while points that fall below the dotted line indicate compounds that caused suppression of the fentanyl signal. Perhaps the most important of the three plots is the response of fentanyl in the presence of heroin (Fig. 2a). No decrease was observed in the peak areas for 1 ng wipe^−1^ fentanyl deposits in the presence of up to 1000 ng wipe^−1^ of heroin (a 0.1% fentanyl mixture by mass). The stability of the fentanyl signal is promising as it shows that fentanyl can be detected in a heroin-fentanyl mixture regardless of the relative percentages of the two compounds. This data also indicates that the proton affinity for fentanyl is likely higher than that of heroin. More interesting than the heroin mixture response is that of fentanyl in the presence of mannitol (Fig. 2a). With the addition of mannitol, a consistent increase in the fentanyl peak area was observed (up to a factor of 2.5 increase at 1000 ng wipe^−1^ of mannitol). This increase may be attributed to either additional proton donation from mannitol, which has a higher propensity to form deprotonated molecular ions than protonated molecular ions [19] or the neutralization of other charge accepting species that could increase available charge for fentanyl. Converse to mannitol, acetaminophen (Fig. 2a) caused a reduction in fentanyl response beyond a 1:1 mixture, indicating competitive ionization was occurring. Inhibition of the fentanyl signal did increase with increasing amounts of acetaminophen though the signal was never completely quenched. At a 1000:1 acetaminophen: fentanyl ratio, fentanyl was still readily detectable (36% peak area relative to pure fentanyl) indicating that detection of fentanyl in a mixture containing acetaminophen would likely not pose any issues. Addition of quinine and procaine exhibited trends similar to heroin (Fig. S19) with slight decreases observed at the 1000:1 level. Caffeine exhibited a trend similar to acetaminophen, though a less dramatic reduction in signal was observed (50% peak area relative to pure fentanyl at the 1000:1 level).

A second study was completed to evaluate whether or not the more potent fentanyl analogues and opioids (carfentanil, and methyl fentanyl, and U-47700) would have similar responses to fentanyl in the presence of heroin. Fig. 2b depicts the response of these three compounds to increasing amounts of heroin. The response from all compounds remained fairly constant across the range of heroin masses, with 91–102% relative response at the 1000:1 ratio, indicating competitive ionization is not a factor for these compounds regardless of the relative amounts. It is important to note that these experiments were completed using trace levels of material so direct analysis of bulk material may cause different effects. The final study evaluating competitive ionization in binary mixtures was completed by analyzing the remaining fentanyl analogues at a 100:1 ratio of heroin: analogue (100 ng wipe^−1^ heroin and 1 ng wipe^−1^ of the analogue), and comparing the response to the signal of the pure analogue. Fig. 2c shows the relative response of all the analogues in the 100:1 ratio, further highlighting that competitive ionization of any of the compounds with heroin would likely not cause a false negative detection. Butyryl fentanyl was the only compound which exhibited an appreciable loss of signal (35% reduction). The similar behavior between fentanyl and the studied analogues suggests that analogues not examined in this work may exhibit the same behavior in the presence of heroin or excipients.

**Fig. 3:**
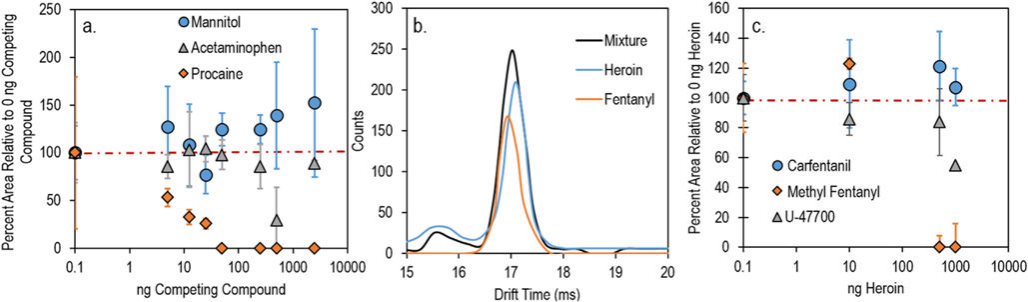
(a) Competitive ionization studies using IMS of fentanyl in the presence of increasing amounts of procaine, mannitol, and acetaminophen. (b) Enlarged overlaid IMS plasmagram windows of the signal for pure heroin, pure fentanyl, and the combination of the two compounds. (c) Competitive ionization studies of select fentanyl analogues in the presence of increasing amounts of heroin. Error bars represent the standard deviation of 3 replicate measurements.

To evaluate competitive ionization effects in the IMS, a parallel study was completed with 5 ng wipe^−1^ used as the constant mass of fentanyl and (0 ng wipe^−1^, 5 ng wipe^−1^, 12.5 ng wipe^−1^, 25 ng wipe^−1^, 50 ng wipe^−1^, 250 ng wipe^−1^, and 500 ng wipe^−1^) as the masses of competing compounds. This provided ratios of competing compound: fentanyl of 0:1, 1:1, 2.5:1, 5:1, 10:1, 50:1, and 100:1. The response of fentanyl, measured as the maximum peak height during analysis, showed little suppression in the presence of acetaminophen (Fig. 3a), while mannitol provided an enhancement in the fentanyl signal, similar to what was observed of caffeine using TD-DART-MS. Caffeine (Fig. S19) also induced minimal suppression for all but the highest level (100:1), where an approximately 50% reduction in the fentanyl signal was observed. Quinine enhanced ionization of fentanyl, with an increase in fentanyl signal observed for all levels (Fig. S19). Procaine proved to be the most problematic excipient, causing little suppression of the fentanyl signal in a 1:1 mixture but complete suppression at higher ratios. Rapid suppression of the fentanyl peak is accompanied by an increasing procaine peak intensity, indicating that procaine has a higher proton affinity and can consume all available charge. This may mean that additional high proton affinity compounds (such as cocaine) could cause similar reductions in fentanyl signals on the IMS when present in a mixture.

Evaluation of IMS competitive ionization effects in fentanyl – heroin mixtures was complicated by resolution limitations of this instrument. A previous study using an earlier model of instrument (Ionscan 400b, Smiths Detection), reported separation of fentanyl and heroin peaks, with detection of both compounds over a wide range of binary compositions [4]. The instrument used in the current study, although a later model, has a lower resolution, and the peaks for the two compounds are not resolved when analyzing mixtures (Fig. 3b). A “combination” peak lies between heroin and fentanyl and has a reduced mobility value of 1.0445 Because alarm windows are typically programed with a window of ±0.003, this combination peak may fall outside of the alarm window of both heroin and fentanyl. This limitation can be addressed by creating a new alarm window for the combination peak. By using three alarm windows (heroin, fentanyl, combination), 100% positive alarms for fentanyl in all heroin: fentanyl combinations (0:1 to 50:1) for 10 ng wipe^−1^ fentanyl were obtained. The alarm for fentanyl was defined as an alarm for either fentanyl and/or the combination peak. Without the combination peak, there were alarms for almost all samples, but typically only for either heroin or fentanyl. Creating additional alarm windows to capture non-resolved and shifted peaks is a workable, but not ideal, solution to the problem. The previous version of the instrument shows that the resolution problems are not a fundamental limitation of IMS, but simply a result of changes in design due to market forces [4]. It is possible to have a system that could resolve fentanyl and heroin combination samples, leading to clear indicators of fentanyl in the presence of heroin.

The same problem with non-resolved peaks occurs for mixtures of heroin with other fentanyl analogues such as methyl fentanyl, o-fluorobutyryl and p-fluoroisobutyryl fentanyl, which are within ±0.037 of the K_0_ value of heroin. The combination peaks that form from these 3 analogues with heroin cannot be reliably differentiated from each other, or from the fentanyl:heroin combination peak. However, a combination peak can be a reliable indicator of the presence of one of the 4 fentanyl compounds in a mixture with heroin. Carfentanil, benzyl, furanyl, acetyl, and valeryl fentanyl have K_0_ values that are further away from heroin, and can be resolved for the entire range of studied compositions (10 ng wipe^−1^ fentanyl analogue combined with 0 ng wipe^−1^, 10 ng wipe^−1^, 500 ng wipe^−1^, and 1000 ng wipe^−1^ of heroin). For these resolved mixtures, ion suppression could be evaluated by monitoring peak intensity, with carfentanil maintaining its intensity even at the highest additions of heroin (Fig. 3c). Detection of U-47700 was also possible across the entire range, and minimal suppression of signal was seen until the highest addition of heroin.

A similar competitive ionization study was completed with buprenorphine and naloxone, the two active ingredients in the drug suboxone^®^, which are present at a 4:1 ratio. To evaluate the competitive ionization that may occur in this mixture, pure buprenorphine and naloxone, as well as a binary mixture, were analyzed using 10 ng wipe^−1^ and 2.5 ng wipe^−1^ deposits respectively. Integrated peak areas of both compounds, when analyzed by TD-DART-MS, increased in the binary mixture, relative to pure, with a 23% increase in signal observed for buprenorphine and 8.5% increase in signal observed for naloxone. When analyzed by IMS, a slight reduction in naloxone signal was observed (approximately 18%) with a slight increase in buprenorphine (approximately 25%) when compared to the pure compounds. Like the fentanyl-heroin binary mixture, these results are promising as they demonstrate that both compounds would be detected in the drug, at least at these lower mass loadings.

### 3.3. Detection in background matrices

The final set of experiments aimed to establish whether or not realistic background matrices would hinder the detection of trace levels of these compounds. In scenarios encountered by first responders or forensic scientists, samples would be collected from materials stored under non-ideal conditions, and therefore would likely contain materials such as phthalates and plasticizers from plastic bags, fingerprint residues, and dirt or dust, in addition to heroin or excipients. To evaluate whether background matrices would complicate detection of these compounds, a binary 10:1 heroin: fentanyl mixture in each of three simulated background matrices was analyzed. Background matrices included artificial fingerprint residue, standard reference material (SRM) sediment to simulate dirt, and retrieval of the materials off of a plastic bag.

**Fig. 4:**
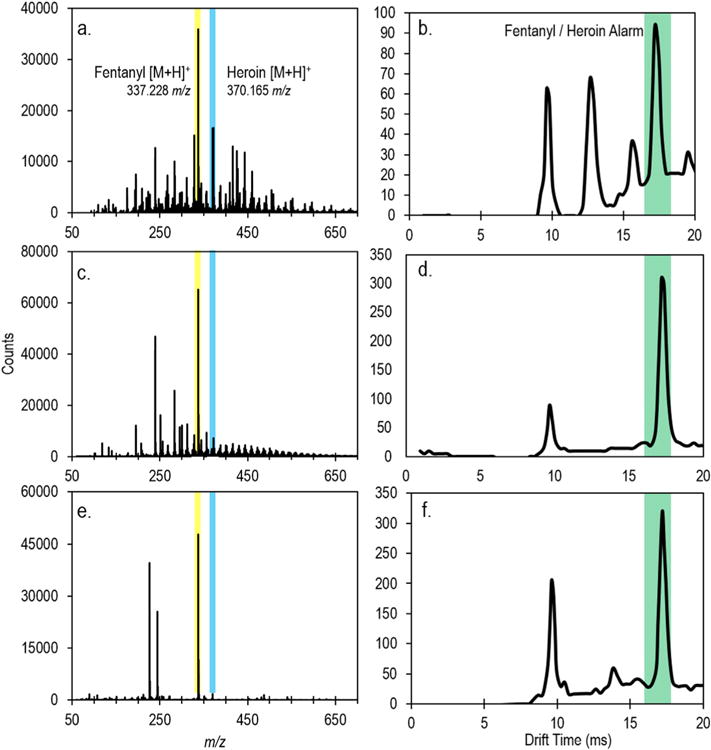
Representative mass spectra (a, c, and e) and averaged IMS spectra (b, d, and f) of fentanyl and heroin in the presence of artificial fingerprint material (a and b), simulant dirt (c and d), and off of a plastic bag (e and f). Spectra are not background subtracted.

The first challenge matrix examined was an artificial fingerprint material containing over forty compounds commonly found in fingerprint residue at biologically relevant concentrations [14]. Approximately 3 μg of the fingerprint material, 10 ng of fentanyl, and 100 ng of heroin were deposited directly onto either the PTFE or meta-aramid wipes and directly inserted into the TD-DART-MS or IMS, respectively. The resulting spectra, shown in Fig. 4a and b, highlight the detection of both compounds in the presence of a largely varied, and complex, matrix. Analysis by IMS produced an alarm for fentanyl, while one of two replicates also produced an alarm for heroin. Cleardown of the TD-DART-MS system after analysis of this background matrix and all others was on the order of 5–20 s. Analysis of the fingerprint residue did increase the IMS cycle time between samples from 15 s to 120 s. Background spectra for this, and all matrices, can be found in the Supplemental information (Fig. S20).

Along with fingerprint residue, it is likely that a surface that is a candidate for bearing trace narcotics will contain dirt or dust. Dirt presents several challenging aspects for trace detection, namely the presence of trace organics, trace inorganics, and particulates which can clog the instrumentation. The effect of dirt on the detection of the binary heroin fentanyl mixture was examined by depositing approximately 50 μL of an aqueous suspension of NIST SRM 1944 (New York/New Jersey Waterway Sediment) onto the wipe along with 100 ng heroin and 10 ng fentanyl followed by direct insertion into the instruments. The aliquot of SRM 1944 was allowed to dry prior to deposition of the heroin and fentanyl. NIST SRM 1944 contains a number of organic and inorganic constituents, as well as particulate with a mean diameter of approximately 150 μm. Fig. 4c highlights the detection of both heroin and fentanyl in this matrix using TD-DART-MS, with a strong signal for fentanyl. The relative signal for heroin is reduced in this matrix, compared to the artificial fingerprint, though it is still detectable, with a signal-to-noise ratio of 127. This reduction in signal for heroin was likely caused by enhanced competitive ionization from a number of compounds in the SRM that have high proton affinities. Analysis by IMS (Fig. 4d) only produced an alarm for fentanyl, and a reduction in the RIP was also observed.

The final background matrix intended to investigate real-world wipe collection and the effects of phthalates and plasticizers on detection by collecting the fentanyl and heroin deposit off of a plastic bag using a wipe. To do so, a polyethylene bag, approximately 7.5 cm by 11.5 cm, was handled for several minutes to purposefully contaminate the bag with real fingerprint residue. After handling, for TD-DART-MS, 100 ng fentanyl and 1 μg heroin were deposited. The entirety of the bag was then wiped and inserted directly into the instrument. The higher levels of fentanyl and heroin were chosen because typical collection efficiencies of the PTFE-coated wipes are on the order of single percentages, which allowed for the collection of approximately 5 ng fentanyl and 50 ng heroin [20]. Fig. 4e shows the rapid detection of both narcotics by TD-DART-MS after being collected off the plastic bag. Again heroin exhibited a suppression in signal, while the signal for fentanyl was quite strong. For IMS, 50 ng of fentanyl and 500 ng of heroin were used because the meta-aramid wipe has an expected collection efficiency around 20% [20]. Both fentanyl and the combination peak were observed and produced alarms (Fig. 4f). Being able to sample rapidly nanogram quantities of material off the outside of a plastic bag, without the need for extraction, is extremely beneficial to first responders and forensic scientists as it provides the opportunity to triage or screen samples without having to open them. This type of approach would minimize risk of exposure to the analyst by reducing the likelihood of coming into contact with powdered material.

## 4. Conclusion

Rapid detection of fentanyl and fentanyl analogues with nanogram to sub-nanogram sensitivity is possible using both TD-DART-MS and IMS. Detection of low levels directly wiped off of a surface can be advantageous to law enforcement, first responders, and forensic scientists as it provides an option for screening an item of evidence without having to come in direct contact with the bulk powder. Both techniques detected fentanyl in the presence of heroin down to 0.1% by weight fentanyl. Lower instrumental resolution in the IMS caused the formation of a heroin and fentanyl combination peak which could be reliably traced to the presence of heroin and fentanyl or a fentanyl analogue. Other adulterants provided varying degrees of signal enhancement or suppression; however, with TD-DART-MS, the fentanyl signal was maintained regardless of excipient and amount present. IMS provided similar results, except with procaine, which consumed all available charge at higher than 1:1 ratios of procaine to fentanyl. Complex matrices, including artificial fingerprint residue, dirt simulant, and a plastic bag, did not prevent detection of fentanyl on either system. The 16 fentanyl analogues behaved similarly to fentanyl, and it is expected comparable results would be obtained in the presence of excipients or background matrices.

While these techniques are not quantitative, they do constitute qualitative tools to help identify and properly mitigate fentanyl and fentanyl analogues in evidence, on persons, and elsewise. Analysis by these techniques is both rapid (less than 10 s), sensitive, and generally discriminative. DART was able to separate all but one pair of isomeric compounds, while IMS had less selectivity in discriminating similar analogues. However, the only identified potential issue for false alarms is the similar K_0_ values for naloxone and despropionyl fentanyl. This work highlights the ability of TD-DART-MS and IMS to rapidly detect fentanyl and fentanyl analogues, though prior work already has highlighted their capabilities in the detection of more conventional narcotics. Both tools provide potential avenues for rapid on-site or laboratory-based screenings from mobile units to forensics laboratories to emergency rooms.

## Supplementary Material

Supp1

## Figures and Tables

**Table 1:** Representative data for all compounds analyzed by IMS and TD-DART-MS. For IMS sensitivities, values with an asterisk (*) are the calculated LOD_90_ value. The average uncertainty in the K_0_ value is ±0.0008.

Compound			IMS		TD-DART-MS			
Name	Molecular Weight (Da)	Molecular Formula	K_0_-value	Sensitivity (ng)	Base Peak + 30 V (*m*/*z)*	Base Peak +90 V (*m*/*z*)	LOD_90_ (ng)	90% Upper Confidence Limit (ng)
*Fentanyls*								
Acetyl Fentanyl	322.204	C_21_H_26_N_2_O	1.0797	5.0	323.212 [M+H]^+^	105.070 [C_8_H_9_]^+^	0.222	0.452
Acryl Fentanyl	334.204	C_22_H_26_N_2_O	1.0569	1.0	335.212 [M+H]^+^	105.070 [C_8_H_9_]^+^	0.145	0.274
Benzyl Fentanyl	322.204	C_21_H_26_N_2_O	1.0817	1.0	323.212 [M+H]^+^	91.055 [C_7_H_7_]^+^	0.0864	0.152
Butyryl Fentanyl	350.236	C_23_H_30_N_2_O	1.0179	5.0	351.244 [M+H]^+^	188.144 [C_13_H_18_N]^+^	0.140	0.259
Carfentanil	394.226	C_24_N_30_N_2_O_3_	0.9712	5.0	395.233 [M+H]^+^	113.084 [C_6_H_11_NO]^+^	0.197	0.392
Cyclopentyl Fentanyl	376.251	C_25_H_32_N_2_O	0.9757	1.0	377.259 [M+H^]+^	188.144 [C_13_H_18_N]^+^	0.165	0.318
Despropionyl Fentanyl	298.184	C_19_H_23_FN_2_	1.1437	1.0	299.192 [M+H]^+^	105.070 [C_8_H_9_]^+^	0.160	0.306
Fentanyl	336.220	C_22_H_28_N_2_O	1.0489	1.401*	337.228 [M+H]^+^	105.070 [C_8_H_9_]^+^	0.142	0.268
*ortho*-Fluorobutyryl Fentanyl	368.226	C_23_H_29_FN_2_O	1.0112	5.0	369.234 [M+H]^+^	105.070 [C_8_H_9_]^+^	0.296	0.657
*para*-Fluoroisobutyryl Fentanyl	368.226	C_23_H_29_FN_2_O	1.0020	1.0	369.234 [M+H]^+^	105.070 [C_8_H_9_]^+^	0.351	0.836
Furanyl Fentanyl	374.199	C_24_H_26_N_2_O_2_	1.0003	5.0	375.207 [M+H]^+^	105.070 [C_8_H_9_]^+^	0.199	0.397
β-Hydroxythiofentanyl	358.171	C_20_H_26_N_2_O_2_S	1.0291	10.0	359.179 [M+H]^+^	192.085 [C_11_H_14_NS]^+^	0.292	0.647
Isobutyryl Fentanyl	350.236	C_23_H_30_N_2_O	1.0195	5.0	351.244 [M+H]^+^	188.144 [C_13_H_18_N]^+^	0.183	0.357
4-Methoxy Butyryl Fentanyl	380.246	C_24_H_32_N_2_O_2_	0.9595	10.0	381.254 [M+H]^+^	188.144 [C_13_H_18_N]^+^	0.101	0.180
4-Methoxy Fentanyl	366.231	C_23_H_30_N_2_O_2_	0.9861	5.0	367.238 [M+H]^+^	188.144 [C_13_H_18_N]^+^	0.262	0.563
*trans*-3-Methyl Fentanyl	350.236	C_23_H_30_N_2_O	1.0217	5.0	351.244 [M+H]^+^	202.160 [C_14_H_20_N]^+^	0.144	0.273
Valeryl Fentanyl	364.251	C_24_H_32_N_2_O	0.9866	5.0	365.259 [M+H]^+^	105.070 [C_8_H_9_]^+^	0.0807	0.142
*Other Opioids*								
Buprenorphine	467.304	C_29_H_41_NO_4_	0.9050	3.857*	468.311 [M+H]^+^	450.301 [M^−^OH]^+^	0.473	1.12
Heroin	369.158	C_21_H_23_NO_5_	1.0386	1.080*	370.165 [M+H]^+^	370.165 [M+H]^+^	2.55	4.11
Methadone	309.209	C_21_H_27_NO	1.1004	0.846*	310.217 [M+H]^+^	265.159 [M^−^C_2_H_6_N]^+^	0.0529	0.0887
Naloxone	327.147	C_19_H_21_NO_4_	1.1425	0.895*	328.155 [M+H]^+^	310.144 [M^−^OH]^+^	0.0930	0.165
U-47700	328.111	C_16_H_22_Cl_2_N_2_O	1.0910	0.5	329.119 [M+H]^+^	172.956 [C_7_H_3_Cl_2_O]+	0.308	0.677

## Appendix A. Supplementary data

Supplementary data associated with this article can be found, in the online version, at http://dx.doi.org/10.1016/j.forc.2017.04.001.
