# Supplementary material for: Rapid detection of fentanyl, fentanyl analogues, and opioids for on-site or laboratory based drug seizure screening using thermal desorption DART-MS and ion mobility spectrometry
Source: Forensic Chem. Author manuscript; Available in PMC 2017 Dec 15. (PMC5731661; doi:10.1016/j.forc.2017.04.001)
Supplement: Supp1 [file NIHMS923556-supplement-Supp1.docx]

Supplemental Information for:

Rapid Detection of Fentanyl, Fentanyl Analogues, and Opioids for on-Site or Laboratory Based Drug Seizure Screening using Thermal Desorption DART-MS and Ion Mobility Spectrometry

Edward Sisco, Jennifer Verkouteren, Jessica Staymates, and Jeffrey Lawrence

**Inkjet Printing Parameters:**

Samples were prepared using a customized drop-on-demand piezoelectric inkjet printer^1, 2^ (Jetlab 4 XL-B, MicroFab Technologies, Inc., Plano, TX) and techniques_3_ described previously in greater detail. Samples were printed from either acetonitrile or methanol based solutions (Cerilliant, Round Rock, Texas) onto woven meta-aramid fiber substrates (Nomex manual swab, Smiths Detection, Danbury, CT). A driving waveform, tuned to match the rheological properties of the printing solvent, was used to eject bursts of drops from a dispensing device (MJ-AB-01-xx-8MX, MicroFab Technologies, Inc.) owning a 50 µm nominal diameter orifice. Droplets were deposited in an array format, where each array spot consisted of a number of drops equivalent to the burst size. To achieve the desired sample loading mass, array (1×1, 2×2, 3×3, 4×4) and burst (1 drop – 999 drops) sizes were adjusted to compensate for drop mass and solution concentration.

**Table 1.** Wave type and nominal parameters for the waveforms used to produce test samples.

Pre- and post-print droplet masses were measured gravimetrically using an integrated microbalance (SE2-F, Sartorius Group, Bohemia, NY) and evaluated for consistency. The population of droplets was required to maintain a relative standard deviation no greater than 1% for burst sizes in excess of 10 drops and 5% for bursts of 10 drops or less. If these requirements were not met the samples were discarded.

**
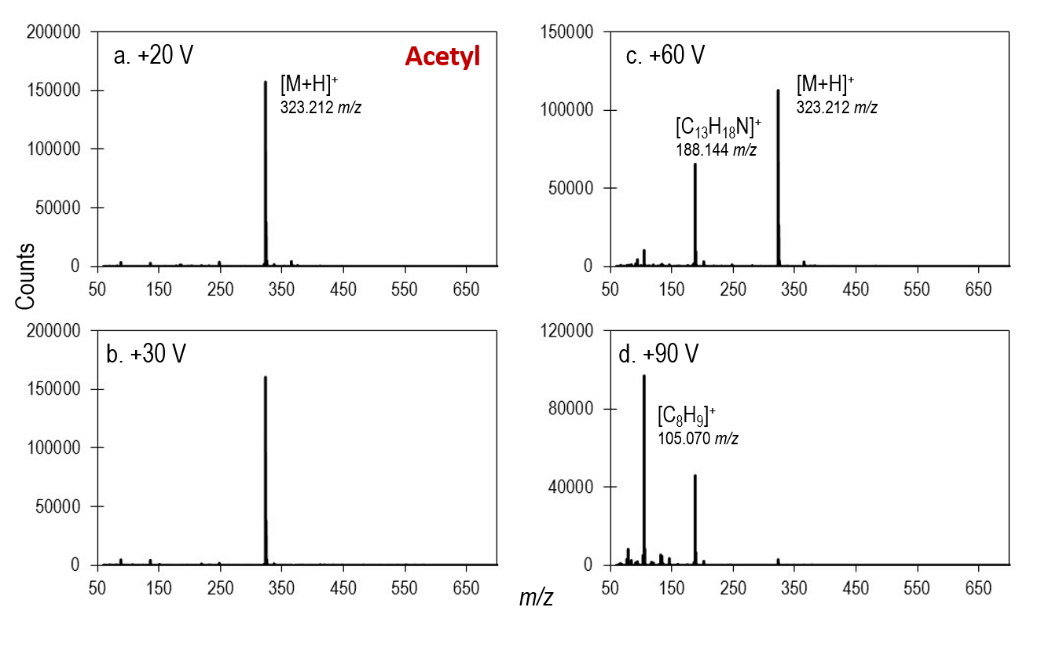
**

**Figure S1.** Representative TD-DART-MS spectra of a 100 ng deposit of acetyl fentanyl at +20 V (a.), +30 V (b.), +60 V (c.), and +90 V (d.) first orifice voltage.

**
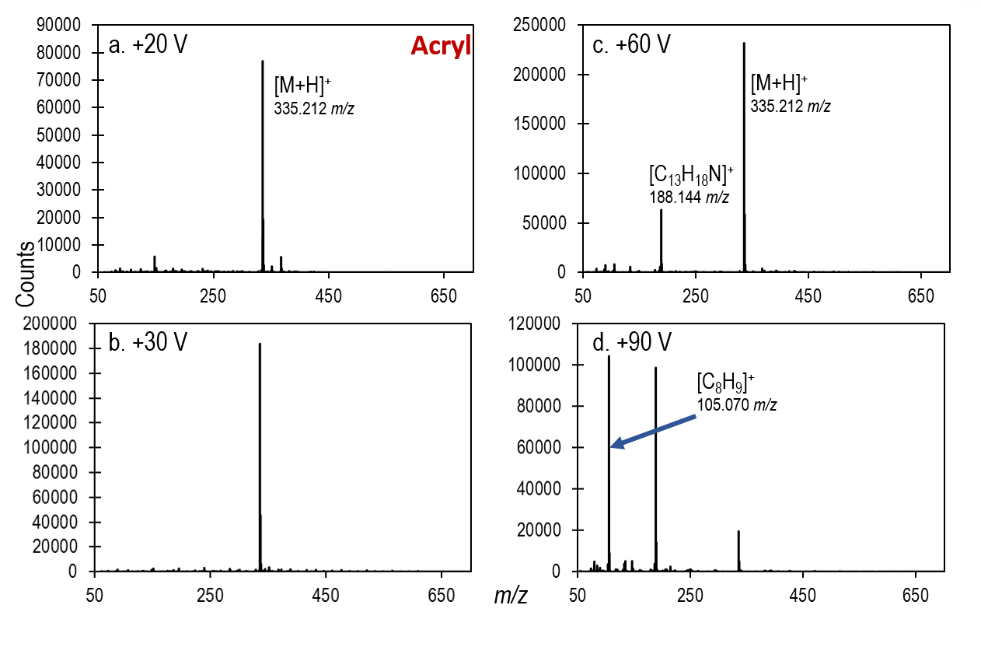
**

**Figure S2.** Representative TD-DART-MS spectra of a 100 ng deposit of acryl fentanyl at +20 V (a.), +30 V (b.), +60 V (c.), and +90 V (d.) first orifice voltage.


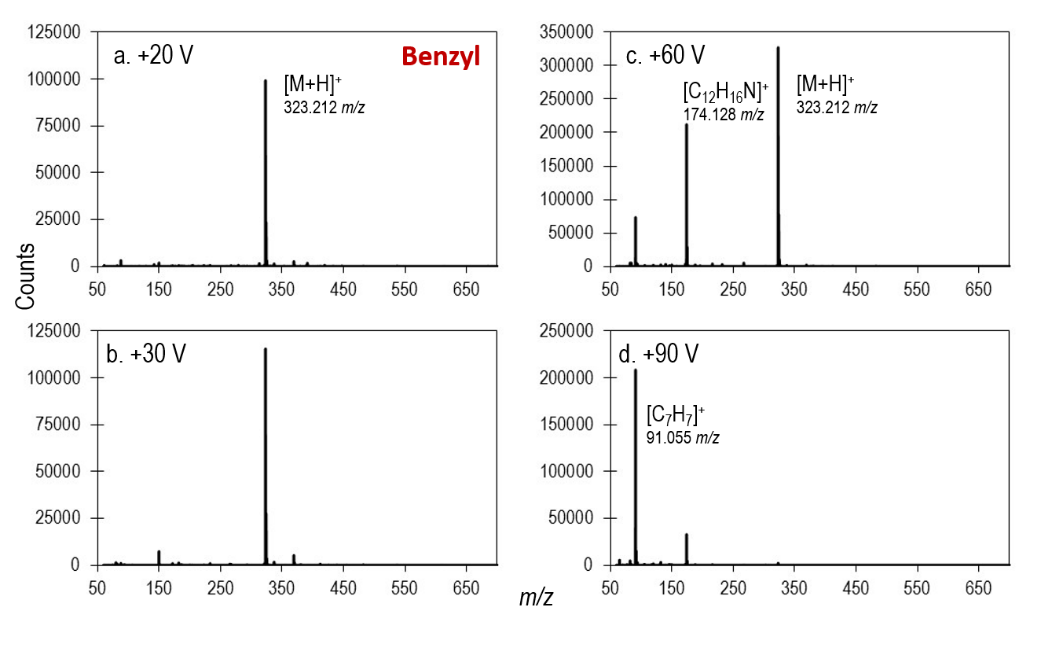


**Figure S3.** Representative TD-DART-MS spectra of a 100 ng deposit of benzyl fentanyl at +20 V (a.), +30 V (b.), +60 V (c.), and +90 V (d.) first orifice voltage.


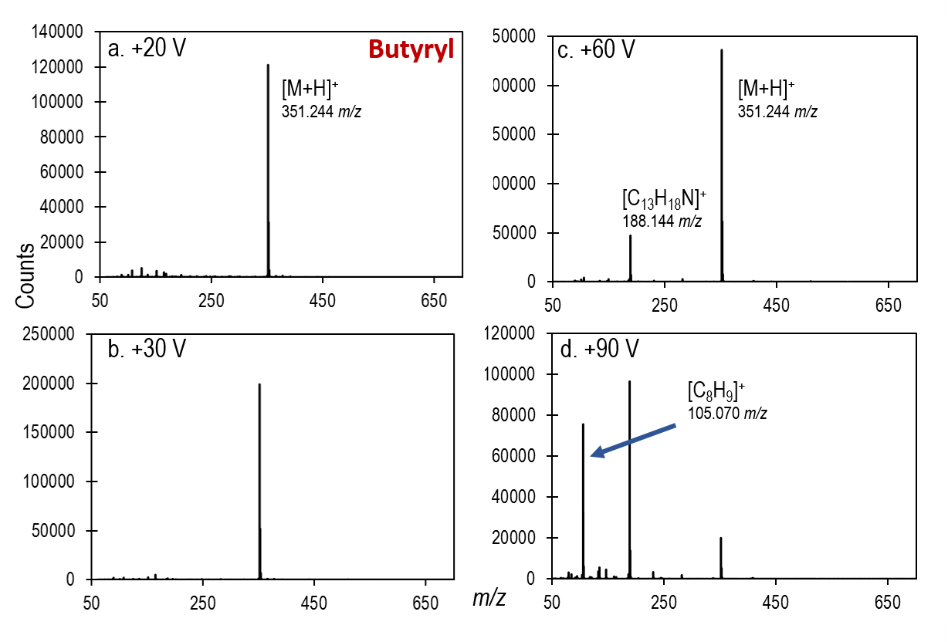


**Figure S4.** Representative TD-DART-MS spectra of a 100 ng deposit of butyryl fentanyl at +20 V (a.), +30 V (b.), +60 V (c.), and +90 V (d.) first orifice voltage.

**
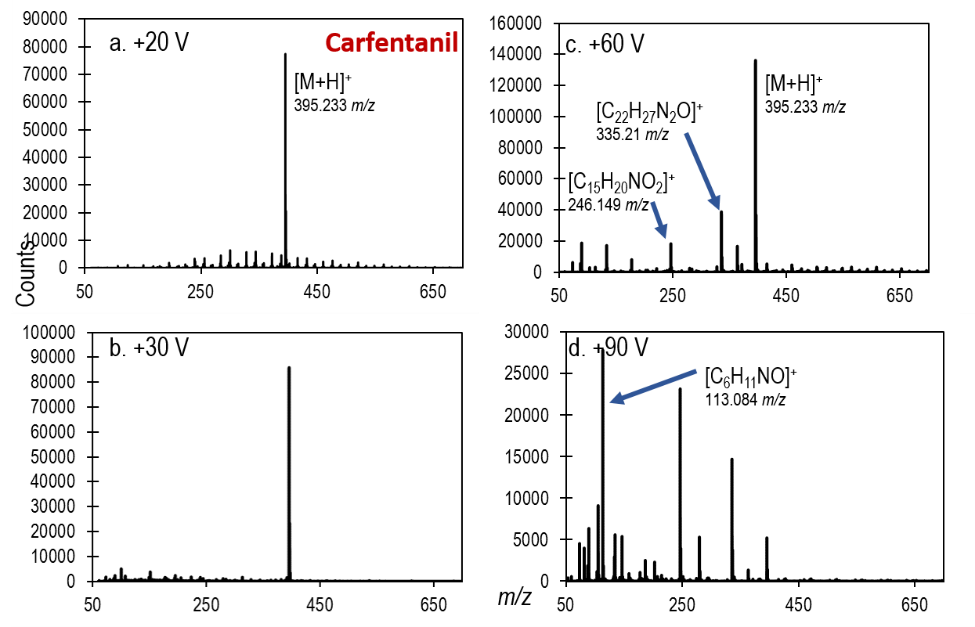
**

**Figure S5.** Representative TD-DART-MS spectra of a 100 ng deposit of carfentanil at +20 V (a.), +30 V (b.), +60 V (c.), and +90 V (d.) first orifice voltage.

**
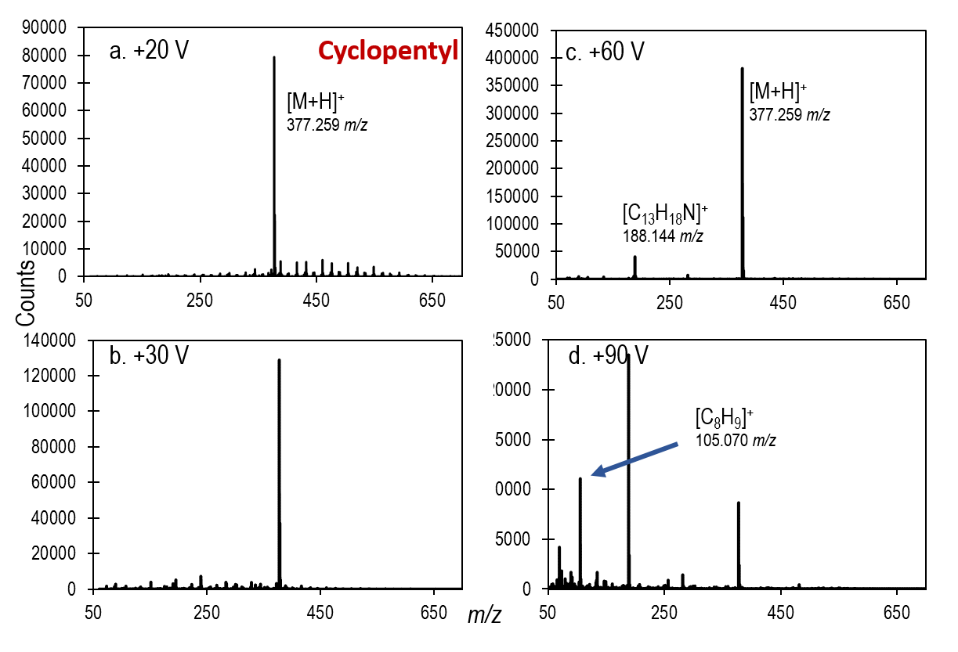
**

**Figure S6.** Representative TD-DART-MS spectra of a 100 ng deposit of cyclopentyl fentanyl at +20 V (a.), +30 V (b.), +60 V (c.), and +90 V (d.) first orifice voltage.


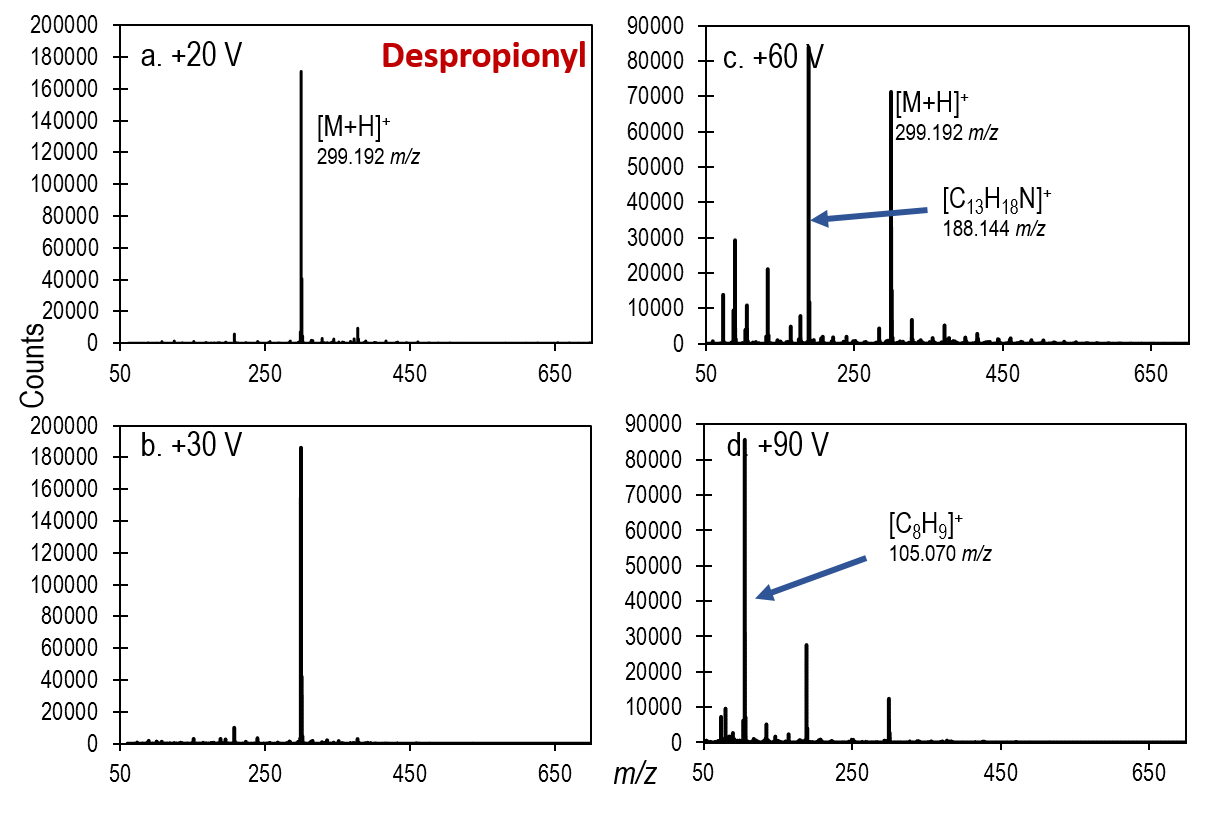


**Figure S7.** Representative TD-DART-MS spectra of a 100 ng deposit of despropionyl fentanyl at +20 V (a.), +30 V (b.), +60 V (c.), and +90 V (d.) first orifice voltage.


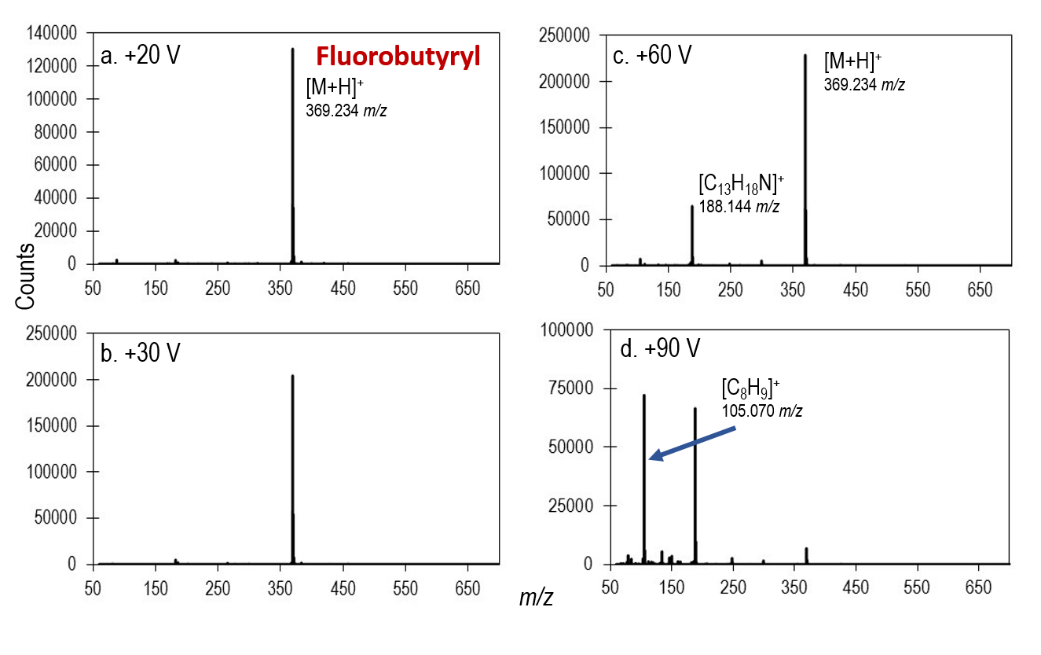


**Figure S8.** Representative TD-DART-MS spectra of a 100 ng deposit of ortho-fluorobutyryl fentanyl at +20 V (a.), +30 V (b.), +60 V (c.), and +90 V (d.) first orifice voltage.


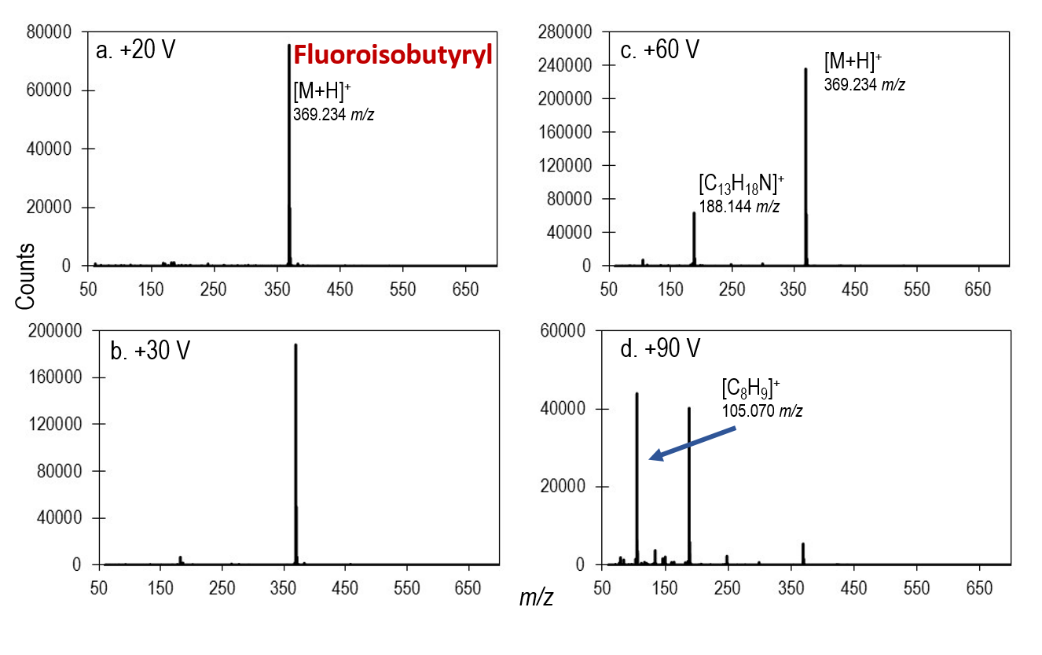


**Figure S9.** Representative TD-DART-MS spectra of a 100 ng deposit of p-fluoroisobutyryl fentanyl at +20 V (a.), +30 V (b.), +60 V (c.), and +90 V (d.) first orifice voltage.


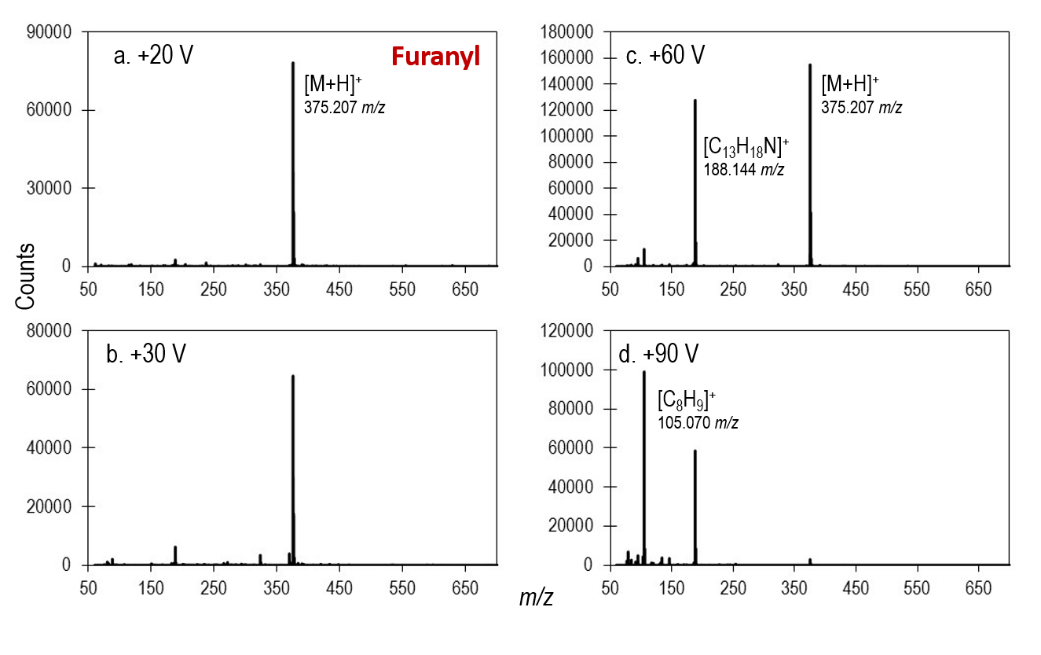


**Figure S10.** Representative TD-DART-MS spectra of a 100 ng deposit of furanyl fentanyl at +20 V (a.), +30 V (b.), +60 V (c.), and +90 V (d.) first orifice voltage.


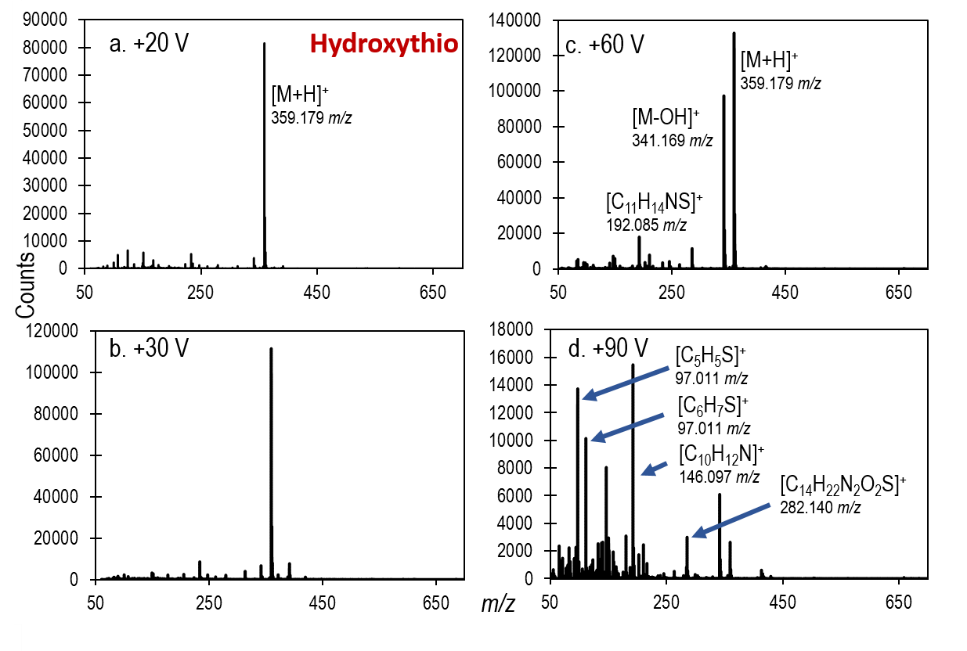


**Figure S11.** Representative TD-DART-MS spectra of a 100 ng deposit of β-hydroxythiofentanyl at +20 V (a.), +30 V (b.), +60 V (c.), and +90 V (d.) first orifice voltage.


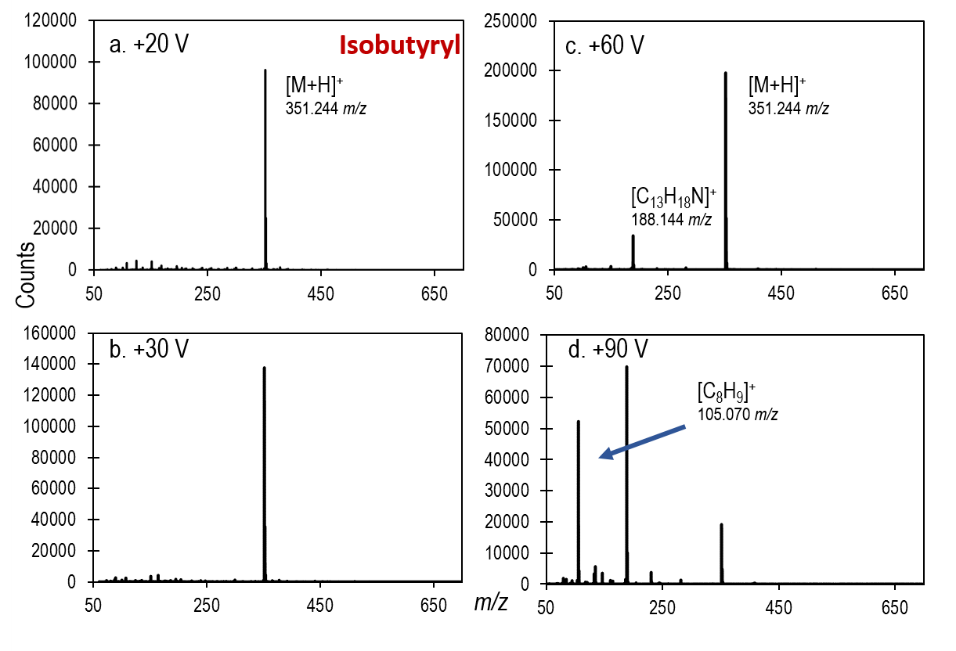


**Figure S12.** Representative TD-DART-MS spectra of a 100 ng deposit of isobutyryl fentanyl at +20 V (a.), +30 V (b.), +60 V (c.), and +90 V (d.) first orifice voltage.


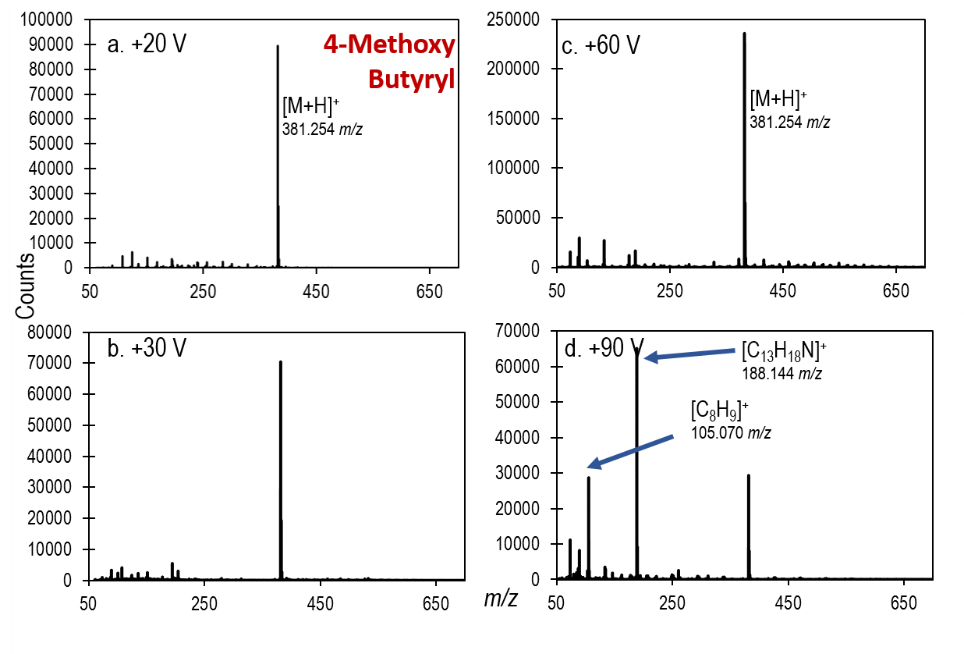


**Figure S13.** Representative TD-DART-MS spectra of a 100 ng deposit of 4-methoxy butyryl fentanyl at +20 V (a.), +30 V (b.), +60 V (c.), and +90 V (d.) first orifice voltage.


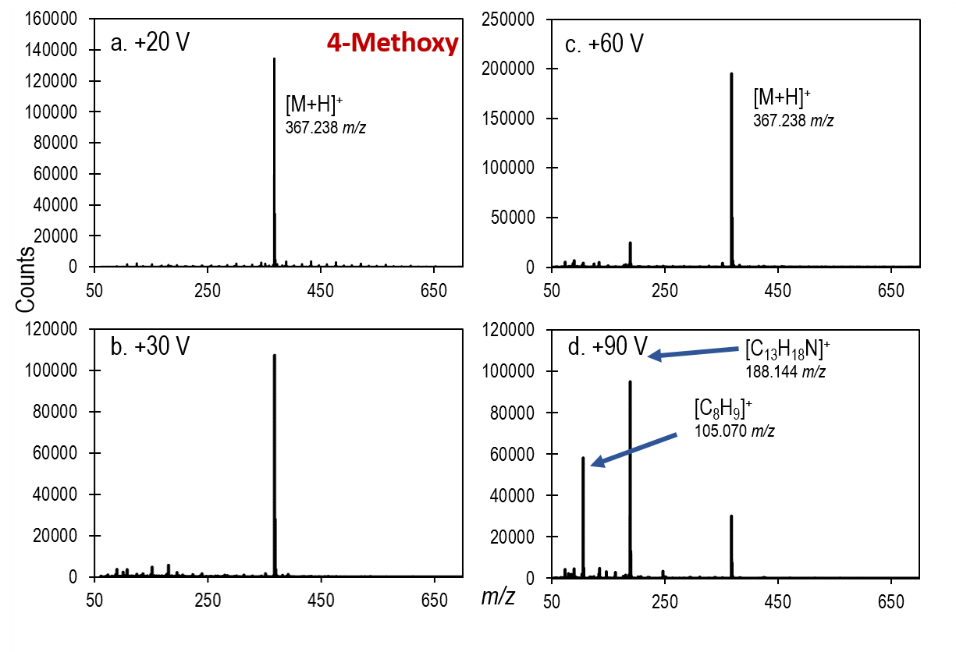


**Figure S14.** Representative TD-DART-MS spectra of a 100 ng deposit of 4-methoxy fentanyl at +20 V (a.), +30 V (b.), +60 V (c.), and +90 V (d.) first orifice voltage.


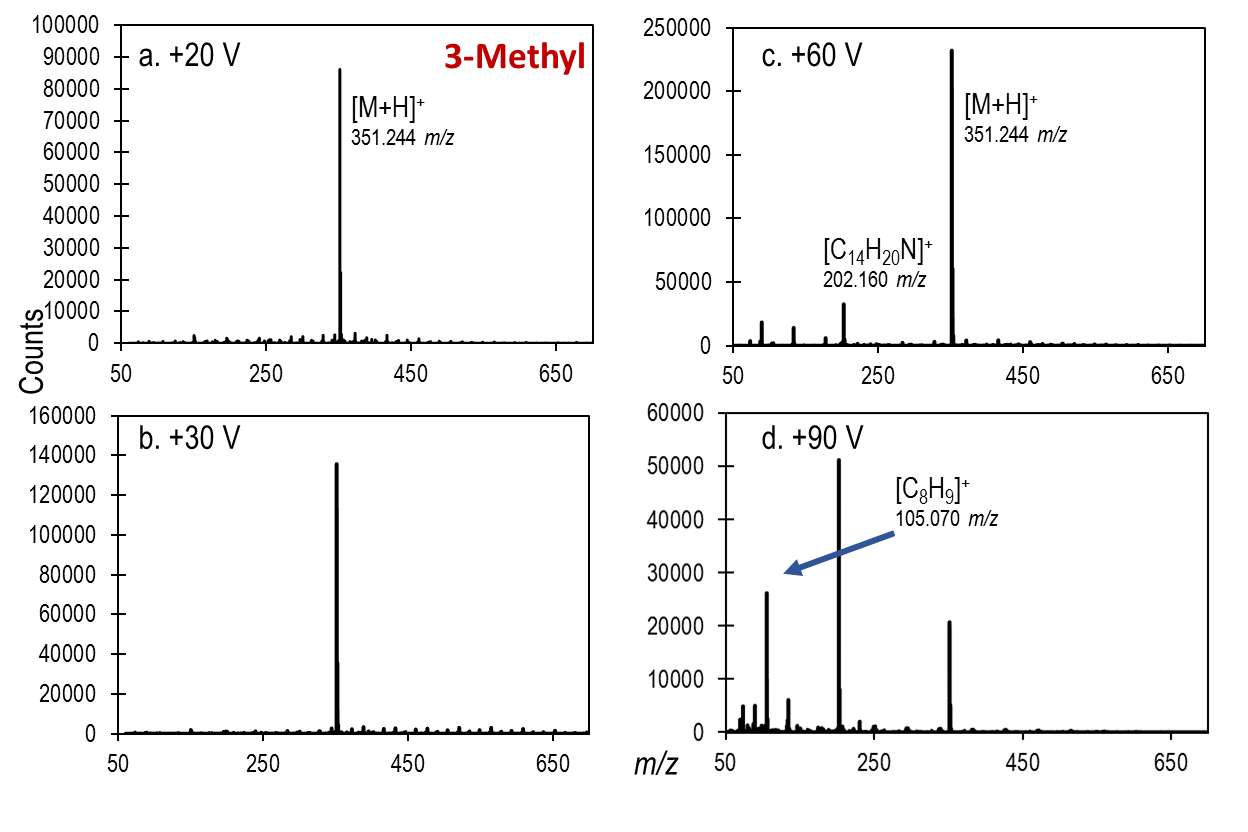


**Figure S15.** Representative TD-DART-MS spectra of a 100 ng deposit of trans-3-methyl fentanyl at +20 V (a.), +30 V (b.), +60 V (c.), and +90 V (d.) first orifice voltage.


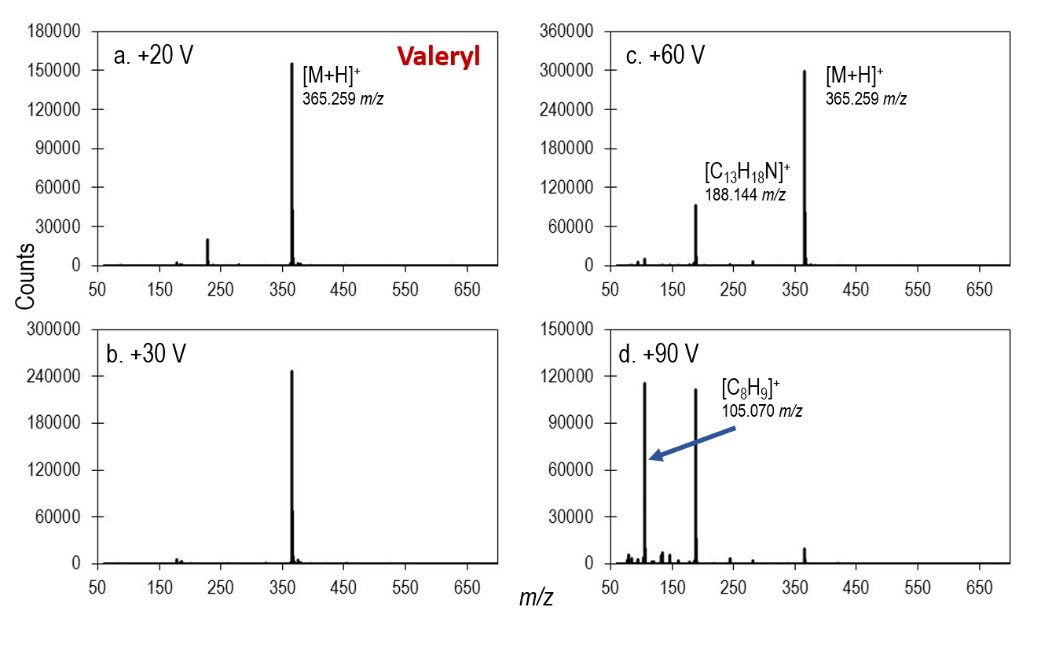


**Figure S16.** Representative TD-DART-MS spectra of a 100 ng deposit of valeryl fentanyl at +20 V (a.), +30 V (b.), +60 V (c.), and +90 V (d.) first orifice voltage.

**
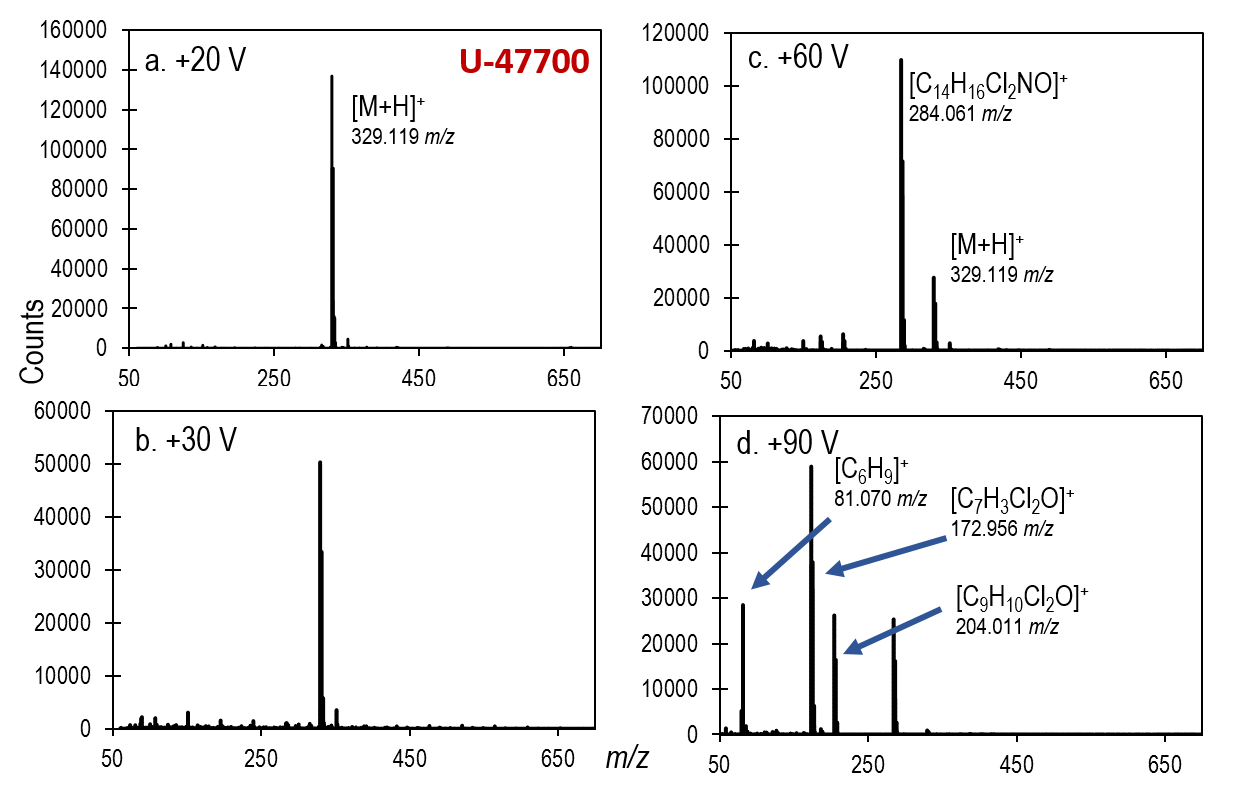
**

**Figure S17.** Representative TD-DART-MS spectra of a 100 ng deposit of U-47700 at +20 V (a.), +30 V (b.), +60 V (c.), and +90 V (d.) first orifice voltage.

**
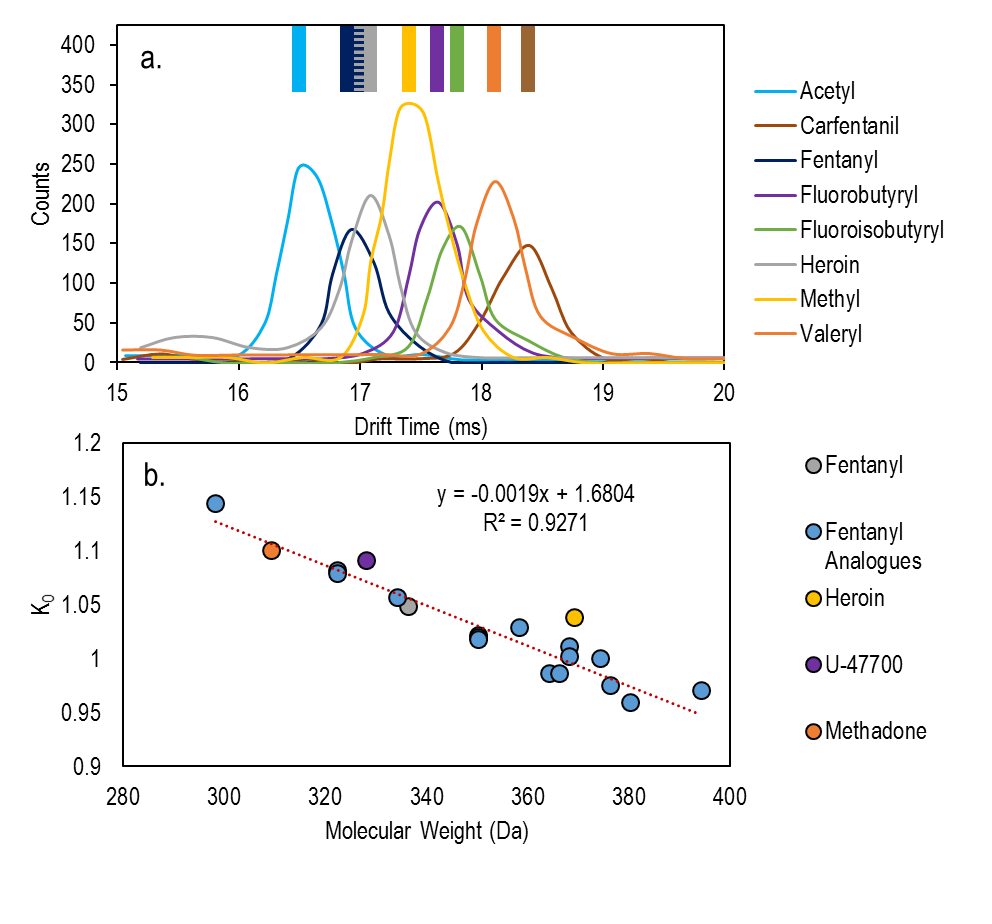
**

**Figure S18.** (a.) Representative IMS response for select fentanyl analogues. These traces are overlays of individual traces from single-component runs. Alarm windows are shown above the plot, with the heroin / fentanyl combination alarm shown in navy and grey lines. (b.) A plot of K_0_ values vs. molecular weight of fentanyl, fentanyl analogues, and associated opioids.


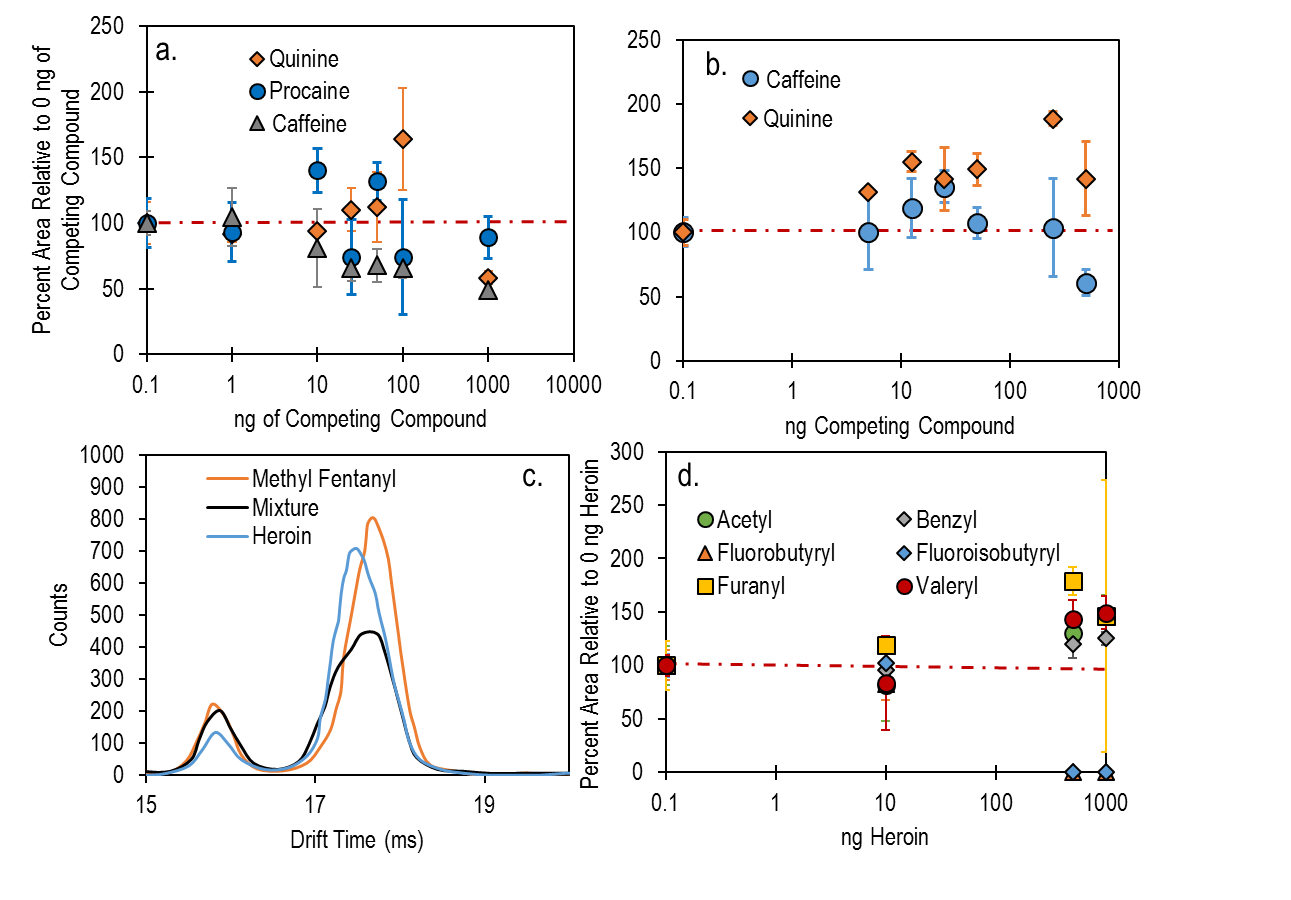


**Figure S19.** (a.) Competitive ionization studies using TD-DART-MS of fentanyl in the presence of increasing amounts of quinine, procaine, and caffeine. (b.) Competitive ionization studies using IMS of fentanyl in the presence of increasing amounts of quinine and caffeine. (c.) Enlarged plasmagram of a heroin / methyl fentanyl mixture showing individual segments where heroin (blue) and methyl fentanyl (orange) are detected, as well as the average plasmagram for the entire analysis (black). (d.) Competitive ionization studies using IMS of fentanyl analogues in the presence of increasing amounts of heroin.


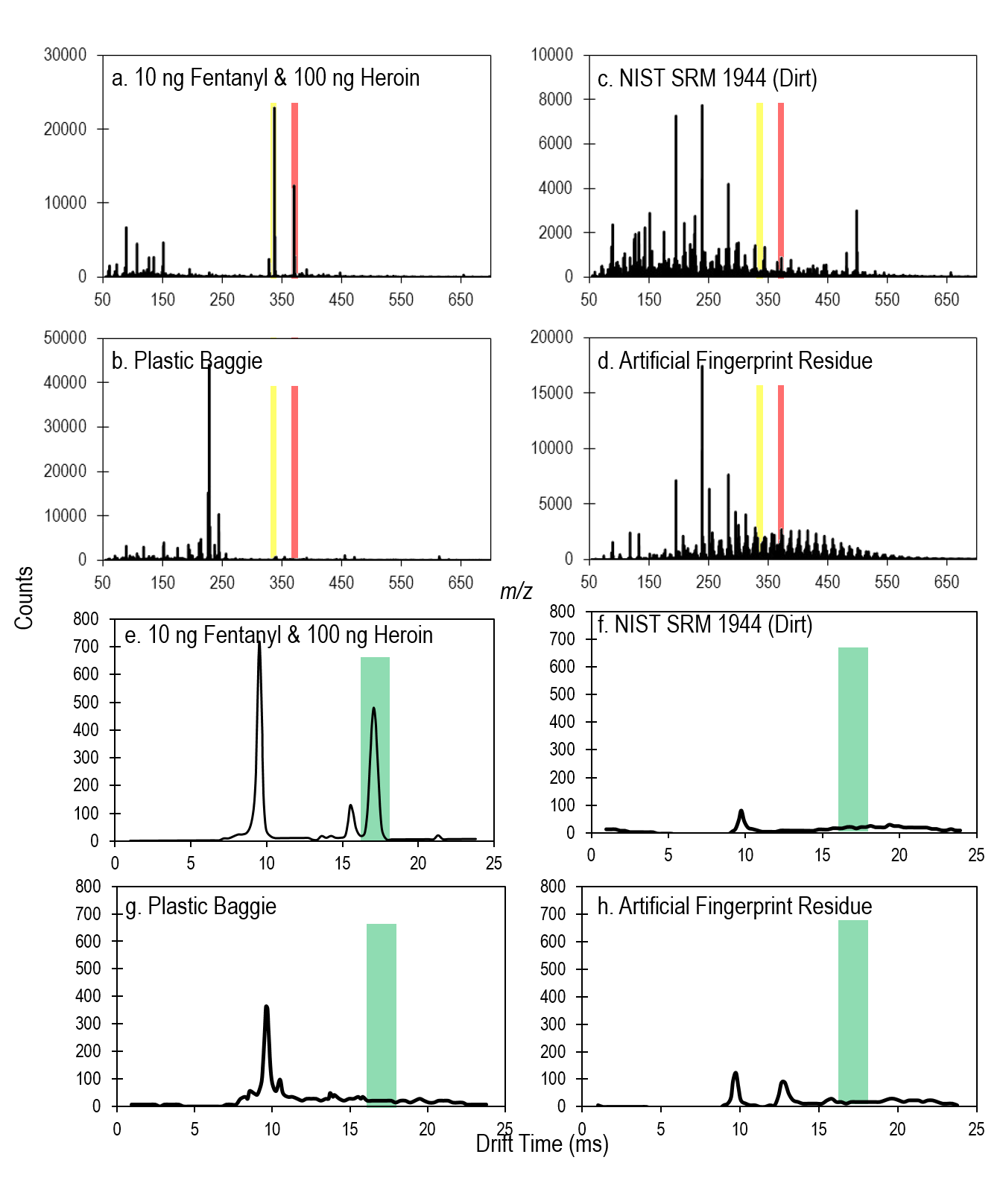


**Figure S20.** Spectra of the 10 ng fentanyl / 100 ng heroin mixture with a complex background using TD-DART-MS (a.) and IMS (e.). Background spectra of the plastic baggie (b. and g.), NIST SRM 1944 dirt (c. and f.), and the artificial fingerprint residue (d. and h.) using TD-DART-MS and IMS respectively.
